# Traditional and Modern Diagnostic Approaches in Diagnosing Pediatric *Helicobacter pylori* Infection

**DOI:** 10.3390/children9070994

**Published:** 2022-07-01

**Authors:** Cristina Oana Mărginean, Lorena Elena Meliț, Maria Oana Săsăran

**Affiliations:** 1 Department of Pediatrics I, George Emil Palade University of Medicine, Pharmacy, Science, and Technology of Târgu Mureș, Gheorghe Marinescu Street No. 38, 540136 Targu Mures, Romania; oana.marginean@umfst.ro; 2 Department of Pediatrics III, George Emil Palade University of Medicine, Pharmacy, Science, and Technology of Târgu Mureș, Gheorghe Marinescu Street No. 38, 540136 Targu Mures, Romania; maria-oana.marginean@umfst.ro

**Keywords:** traditional and modern diagnostic approaches, *H. pylori* infection, children

## Abstract

*Helicobacter pylori* (*H. pylori*) is the most common bacterial infection worldwide, is usually acquired during childhood and is related to gastric carcinogenesis during adulthood. Therefore, its early proper diagnosis and subsequent successful eradication represent the cornerstones of gastric cancer prevention. The aim of this narrative review was to assess traditional and modern diagnostic methods in terms of *H. pylori* diagnosis. Several invasive and non-invasive methods were described, each with its pros and cons. The invasive diagnostic methods comprise endoscopy with biopsy, rapid urease tests, histopathological exams, cultures and biopsy-based molecular tests. Among these, probably the most available, accurate and cost-effective test remains histology, albeit molecular tests definitely remain the most accurate despite their high costs. The non-invasive tests consist of urea breath tests, serology, stool antigens and non-invasive molecular tests. Urea breath tests and stool antigens are the most useful in clinical practice both for the diagnosis of *H. pylori* infection and for monitoring the eradication of this infection after therapy. The challenges related to accurate diagnosis lead to a choice that must be based on *H. pylori* virulence, environmental factors and host peculiarities.

## 1. Introduction

*Helicobacter pylori* (*H. pylori*), a spiral-shaped gram-negative bacteria previously named *Campylobacter pylori*, was thoroughly described in 1982 by Robin Warren and Barry J. Marshall, who received the Nobel Prize in Medicine in 2005 for defining its role in the etiopathogenesis of gastro-duodenal ulcers [1]. The *H. pylori* surface is coated by thermal shock protein and urease [2]. *H. pylori* flagella not only increase their mobility but also have essential roles in initiating chemotaxis and biofilm formation, triggering gastric inflammation and enabling immune evasion [3,4,5]. Urease is an important adjuvant factor for bacterial colonization since it breaks urea into ammonia such that the increase in gastric pH required for bacterial survive in the gastric microenvironment can be determined [6]. Other virulence factors such as vacuolating cytotoxin (VacA) or cytotoxin-associated antigen (CagA) make major contributions to the development of *H. pylori* chronic gastritis, which is a complex process involving also the contribution of the host’s immune responses [7]. Vac A is expressed by approximately 50% of *H. pylori* strains in its mature form and enables the synthesis of proinflammatory cytokines, also facilitating chronic colonization of the gastric mucosa [8]. In addition, VacA is able to change the structure of anions within endosomes, causing osmotic edema and subsequent apoptosis in the gastric epithelium [9,10]. CagA, probably the most important virulence factor of *H. pylori*, if present, contributes to the activation of certain proinflammatory pathways such as NF-kB, resulting in severe inflammatory responses, but at the same time, it favors the production of catalase, which enhances the survival of *H. pylori* within the host’s gastric microenvironment by hindering the formation of reactive oxygen compounds from hydrogen peroxide [11]. Aside from these two major virulence factors of *H. pylori*, a wide spectrum of adhesins have been discovered, including SabA (sialic acid-binding adhesin), BabA (blood-group-antigen-binding adhesin), AlpA/B (adherence-associated lipoprotein A and B) and outer inflammatory protein A (OipA), which are all involved in mediating the adherence and binding of this bacterium to the gastric cell receptors [12,13,14,15,16]. Further studies proved that, in addition to the aforementioned virulence factors, the type IV secretion system suppresses phagocytosis, phospholipases favor the degradation of several lipids and injures the gastric mucus layer, gamma-glutamyl transpeptidase triggers the apoptosis and necrosis of dendritic cells, arginase also induces apoptosis and hinders bacterial death, neutrophil-activating protein enables neutrophil adherence to gastric epithelial cells, superoxidase dismutase promotes colonization and provides a shield against the action of reactive oxygen species, catalase determines mutagenesis, and cholesteryl-α-glucosyltransferase decreases both phagocytic activity and immune responses [3,4,5,17,18,19,20]. Nevertheless, VacA and CagA remain the most important virulence factors since VacA was related to an increased risk for carcinogenesis, while CagA was associated with ulcer disease, and according to their expression, *H. pylori* strains vary from reduced virulence to high virulence strains [21].

At the opposite end is the host immune system, which is activated by the colonization of the stomach and the virulence factors that are released into the host cells in order to trigger a strong immune response via innate immune receptors defined as Toll-like receptors with further crucial implications in the expression of proinflammatory cytokines [22]. Unfortunately, the activation of immune responses worsens oxidative stress, resulting in cellular damage and eventually promoting the complex pathway towards gastric carcinogenesis [23]. As a result of immune responses, increased levels of TGF-β, IL-1β, IL-8 and IL-18 along with a higher number of macrophages can be found within the gastric mucosa [7]. All of these cytokines, especially TGF-β, which is produced excessively by dendritic cells, are involved in regulating local inflammation and enhances the presence of *H. pylori* at this level through the activity of T regulatory cells, with major contributions in reducing inflammation and in increasing bacterial density, eventually promoting chronic infection [24].

Although it is the most common bacterial infection worldwide and is commonly acquired during childhood, affecting approximately 50% of the global population, its incidence depends on the geographical area, varying between 20–50% in developed countries and 80% in developing countries [25,26]. Thus, epidemiological studies proved the prevalence of *H. pylori* infection reaches up to 60% in Asia, Latin America or Africa, whereas in North America and Europe, it accounts for less than 10% [27,28,29]. Moreover, the diagnosis of this infection involves multiple challenges due to several problems related to the diagnostic methods such as invasiveness, lack of sensitivity or specificity, the direct association between accuracy and *H. pylori* incidence or even interobserver-related variability. The clinician should choose their diagnostic methods wisely, assessing all the pros and cons for each approach to provide the highest standard of care.

The aim of this narrative review was to assess the pros and cons in terms of *H.* *pylori* diagnosis. The following keywords were used for the literature search: *H. pylori* children diagnosis, endoscopy, *H. pylori* histology, rapid urease test, culture, molecular tests, urea breath test, stool antigen test and serology.

## 2. Who Should Be Tested, Why and How to Choose the Testing Method?

It is not uncommon for children with *H. pylori* to be asymptomatic, and therefore, why should they be tested and how do we select the ones that require testing? Moreover, which is the most accurate but harmless testing methods in these children? Even more challenging is to decide who should be treated. These are only a few of the debatable topics related to *H. pylori* infection, especially in pediatric patients. Taking into account that, generally, treatment is recommended in all patients with *H. pylori* gastritis, even in those without symptoms [30,31], it is extremely important to choose the most appropriate diagnostic method. According to the most recent guidelines [32], the recommendations for *H. pylori* testing focus on subjects with dyspepsia originating from geographic areas with high prevalence; those with peptic ulcers, especially in individuals who use aspirin or non-steroidal anti-inflammatory drugs, or those with a history of peptic ulcer; those with gastritis, especially subjects following long-term proton pump inhibitor treatment; subjects with gastric cancer or increased risk of gastric cancer, or those with localized early stage MALToma; and last but not least, people with idiopathic thrombocytopenic purpura, iron deficiency anemia or vitamin B12 deficiency without a probable cause. Most of the extraintestinal manifestations associated with *H. pylori* seem to be related to the systemic subclinical inflammation triggered by infections with *H. pylori*, which is no longer a myth in children [33,34]. Therefore, an early diagnosis is absolutely mandatory for preventing long-term complications.

### 2.1. Traditional and New Diagnostic Tests—Advantages, Disadvantages and Limitations

All of the available tests for *H. pylori* detection are divided into invasive—including endoscopy, histological examinations, rapid urease tests and cultures—as well as non-invasive—comprising urea breath test, stool antigens and serology. In addition, molecular testing based on real-time polymerase chain reaction (PCR) might be either invasive or non-invasive depending on the used clinical sample.

#### 2.1.1. Endoscopy

According to the European Helicobacter Study Group, endoscopy is recommended for patients with dyspepsia below the ages of 45–50 years and its effectiveness has been proven in the lack of other alarming signs and in patients without any symptoms of gastroesophageal reflux [35]. Endoscopy allows for the visual assessment of gastric mucosa, enabling the identification of certain macroscopic abnormalities or suspect lesions, and it provides gastric biopsy samples, which will be extremely useful for other invasive diagnostic methods [32] (Table 1). A proper assessment of *H. pylori*-positive gastritis requires at least six biopsies from different areas of the gastric mucosa including the antrum, gastric corpus, as well as small and large curves. In certain suspicious lesions, it is absolutely mandatory to take several biopsy samples from the lesion. The accuracy of endoscopy in diagnosing *H. pylori* infection is limited by the wide range of macroscopic aspects that can occur as a result of different stages of gastritis varying from active inflammation and atrophy to intestinal metaplasia [35]. It is a well-documented fact that the macroscopic nodular aspect of gastric mucosa is associated with both *H. pylori* infection and the mucosal density of the bacterium [36,37] (Table 1). This finding is supported by studies performed on both adults and children [33,36,37,38]. Thus, nodular gastritis is commonly encountered in children with *H. pylori* infection, and it seems to contribute to the formation of lymphoid follicles and lymphoepithelial lesions, resulting in grades 1 to 5 gastric lymphoid hyperplasia [39]. Nevertheless, several pediatric studies suggested that the prevalence of atrophic gastritis associated with *H. pylori* infection is higher in children and young adults when compared with mucosal nodularity in these age groups [40,41]. Aside from nodularity and atrophy, other macroscopic aspect of the gastric mucosa associated with *H. pylori* infection include mucosal edema, diffuse erythema or hypertrophy of the mucosal folds [42,43]. Certain new endoscopic methods such as linked color imaging and blue laser imaging have been developed to improve the limitations in diagnosis [44,45]. In addition, other innovative endoscopic methods were proposed for increasing the accuracy of biopsy collection [46]. Multiple recent studies underlined the utility of blue laser imaging in diagnosing gastric cancer regardless of the stage and even gastric metaplasia [47,48,49,50,51] (Table 1).

Narrow band imaging (NBI) is a more specific endoscopy-based diagnostic tool that is superior to other invasive diagnostic methods due to its capacity to provide a faster diagnosis. NBI is based on the penetration variability of the light waves since it is well-documented that long wavelengths have the ability to penetrate deeper into the tissue, while short wavelengths remain superficial. Thus, short-wavelength light increases the visualization of contrast areas on the gastric epithelial layer due to its reflection and better spread [52,53]. In addition, a study performed on children proved that, despite its low specificity, NBI is useful for identifying the most-likely areas to be infected, indicating the optimal areas for biopsy [38]. It was also stated that histopathological severity is correlated with an endoscopic view provided by this technique [53] (Table 1).

Aside from all of the previously mentioned image-enhanced endoscopy, magnifying endoscopy has emerged as a new technique based on this endoscopic method and allows for the prediction of *H. pylori* presence based on an assessment of the mucosal microvascular architecture. Thus, a ‘pit plus vascular patters’ is a significant sign for *H. pylori* infection identified via magnifying endoscopy [54]. A recent meta-analysis that aimed to assess the diagnostic accuracy of magnifying endoscopy in predicting *H. pylori* infection underlined that this method is accurate for detecting *H. pylori* in both chromoendoscopy and white-light [55] (Table 1).

One of the most important limitations of endoscopy and endoscopic views is the interobserver or intraobserver variability augmented by the lack of objective indicators [56] (Table 1). A very recent study indicated that this risk might be mitigated using artificial intelligence, which might be helpful for image recognition and classification [57]. These findings were previously supported by other studies, which revealed that artificial intelligence-assisted endoscopy could be useful in providing a second opinion by avoiding operator dependency in diagnostic endoscopy [58,59]. This technique using endoscopic imaged proved to be useful in detecting both neoplastic and non-neoplastic lesions in the gastrointestinal tract [60]. The diagnostic performance of artificial intelligence seems to be spared by the influence of either the type of this method or the type of endoscopic images used [57]. Most of the studies with patient-based and image-based analyses suggested a good accuracy of artificial intelligence in diagnosing *H. pylori* infection [43,56,61,62,63,64,65]. Thus, an artificial-intelligence-based algorithm might be useful in providing a fast diagnosis of *H. pylori* infection, which is crucial for the prompt eradication of this infection, preventing further devastating long-term impacts on the gastric mucosa.

#### 2.1.2. The Rapid Urease Test

The Rapid Urease Test (RUT) is an invasive, low cost, rapid and relatively highly specific test that can detect the presence of *H. pylori* after a maximum of 12–24 h from endoscopy based on gastric biopsy. Thus, RUT has the ability to detect urease activity, a well-known enzyme synthetized by *H. pylori*, which will split the urea test reagent, resulting in ammonia with consecutive increases in pH identified by the phenol red indicator [66,67,68].

Taking into account that it is based on detecting urease activity, RUT is prone to both false-negative and false-positive results. False-negative results are more common than false-positive ones, and their exclusive use is not recommended for the exclusion of *H. pylori* infection [32]. Certain conditions were reported to increase the risk for false-negative RUT results such as low bacterial colonization, i.e., less than 10^4^ bacterial cells; the recent use of antibiotics, bismuth, H_2_-receptor agonists and proton pump inhibitors; as well as assessments of gastric biopsy specimens taken from areas of metaplasia, atrophy or recent bleeding gastroduodenal ulcers [66,69,70]. Albeit less common, false-positive reactions are usually the result of other urease-producing bacteria that are present in the stomach such as *Streptococcus salivarius*, *Proteus mirabilis*, *Klebsiella pneumoniae*, *Enterobacter cloacae*, *Citrobacter freundii* or *Staphylococcus capitis* [70,71,72]. The sensitivity rate increases if at least two biopsies are assessed from both the antrum and corpus [73]. Despite all of these inconveniences, RUT remains a relatively specific, reaching up to 95–100%, and moderately sensitive, with a rate of 85–95%, test [66,68] (Table 1).

According to the Maastricht V Consensus Report, a positive RUT can be used for establishing the diagnosis of *H. pylori* infection and for recommending the eradication treatment, but it is not recommendable to assess the eradication of this infection based on a negative RUT, with a supplementary method being required for excluding *H. pylori* [32] (Table 1).

#### 2.1.3. Histopathological Exam

Histological assessment of gastric biopsy when upper endoscopy is required can be recommended for the primary diagnosis of *H. pylori* gastritis [54]. *H. pylori* can be detected from histopathological exams only on well-stained and sufficiently thin sections [74]. Multiple selective stains are used worldwide for the detection of this bacterium on histology such as Giemsa, *H. pylori* silver stain, Warthin-Starry, Dieterle, Gimenez, McMullen, acridine orange and immunostaining, but Giemsa remains the most commonly used due to its low cost, sensitivity, ease of use and reproducibility [54]. Nevertheless, it is generally stated that the Giemsa stain has a lower sensibility rate when compared with hematoxylin-eosin but a higher specificity and a lower false-positive rate, which can be further reduced if immunohistochemistry is used, which definitely represents the most visible and specific staining, but unfortunately, it is not always available [46]. One of the most recently used methods is fluorescent nucleic acid peptide in situ hybridization, with a specificity of 100%, good cost-effectiveness, fast processing time and the capacity to identify undetectable forms in routine staining. Nonetheless, these advantages are shadowed by the laborious preparation time, and the need for a special microscope with fluorescence and an experienced observer [46,75,76,77]. In addition, another novel fluorescence-based method based on a γ-glutamyl transpeptidase (GGT) achievable fluorescent probe is a fast detection tool for *H. pylori* and provides the result in approximately 15 min, but its sensitivity remains poor, reaching only 82% [78] (Table 1).

Certain conditions might decrease the accuracy of histopathological exams including intestinal metaplasia, gastric atrophy, low bacterial density, uneven *H. pylori* distribution on the mucosal surface, long-term proton pump inhibitor use, antibiotics or bismuth administration [54,74,79]. Similar to the RUT, these risks could be avoided if multiple gastric biopsies are taken, not only from the gastric antrum but also especially from the corpus, since it was proved that biopsies from this are extremely valuable for increasing the chance to detect *H. pylori* in patients with a history of atrophic gastritis or long-term proton pump inhibitor treatment [74]. The specificity of the histopathological exam can reach up to 100%, while the sensitivity varies between 50% and 95% depending on the location, quality, size and frequency of the biopsy as well as on the stain used [70,80]. Thus, the updated Sydney System stated that five gastric biopsy specimens are required for a proper assessment of the stage and severity degree of *H. pylori*-positive gastritis [81]. These biopsies should be taken from different areas of the gastric mucosa, as follows: two from the gastric antrum, two from the corpus and one from the incisura angularis (Table 1).

Except for accurately detecting *H. pylori* and assessing the severity of the gastritis, the histopathological exam has a crucial advantage over all other methods in terms of diagnosing precancerous lesions such as atrophic gastritis and intestinal metaplasia, which are well-documented consequences of *H. pylori* infection. Even more challenging is to detect *H. pylori* in these patients carrying these conditions and those with gastric cancer, with the corpus’s greater curvature side being proven as the optimal site for biopsy in these groups of patients [74].

#### 2.1.4. Culture

The cultivation of pathogens from gastric biopsy specimens provides a wide spectrum of information regarding the morphological, biological and biochemical properties of *H. pylori*. Additionally, the identification of a pure culture of *H. pylori* as a result of cultivation allows for both the determination of antibiotic resistance and its close monitoring [66]. This method has a specificity of 100%, but the sensitivity presents a wide variation, between 50 and 90% [74,79]. Nevertheless, false-negative results are possible due to several host-related and environmental factors. Among the host-related factors, we recall alcohol consumption; low bacterial density; upper gastrointestinal hemorrhage; and treatment with proton pump inhibitors, H_2_ receptor antagonists or antibiotics [66]. On the other hand, certain environmental factors were incriminated as contributors to the false-negative results such as delayed transport of biopsy specimens, poor sample quality, inappropriate transportation by exposing the sample to aerobic conditions, inexperience of the microbiologist or issues related to method testing [66] (Table 1).

The high accuracy of this method is hindered by the limitations related to its laborious processing requiring strict transportation conditions for preserving the bacterium in a viable state, microaerophilic conditions with an oxygen content of less than 5%, high costs due to special laboratory equipment and reagents, designated nutrient media and experienced staff [54] (Table 1).

Some of the above disadvantages and limitations could be solved or avoided by recommending the patient to cease the administration of any treatment that might impair the results at least 4 weeks before the upper endoscopy and by taking at two biopsies from the antrum and two from the corpus [54].

Despite the multitude of factors that limit its wide use in clinical practice, cultures remain extremely valuable and, according to the Maastricht V Consensus Report, should be used in geographical areas with a primary resistance to clarithromycin higher than 20%. Based on much evidence, the same experts suggested that this method should be performed in cases where second-line eradication treatment failed in order to properly choose the next antibiotics based on *H. pylori* sensitivity features [32,82,83,84] (Table 1).

#### 2.1.5. Urea Breath Test (UBT)

UBT is a respiratory test based on the *H. pylori* urease activity that converts urea into ammonia, thus neutralizing the gastric acidic pH to enable *H. pylori* to penetrate the mucous layer and to attach itself to the gastric wall cells. Commonly, UBT uses 75 mg of ^13^C. The test involves the administration of radioactively labeled urea and the quantification of the exhaled ^13^C/^14^C before and after swallowing the urea using mass spectrometry. Thus, four samples should be collected from the patient after a digestive rest of at least 6 h: two before urea and two after urea ingestion. In fact, the current pediatric guidelines indicated that fastening periods are optimal at 8–12 h for children and at 4–6 h for infants younger than 6 months [85]. After collecting the first two samples in tubes or bags, the patient will receive a ‘test mass’ followed by a solution containing ^13^C-labeled urea mixed with water. The following two samples will be collected after 30 min from the ingestion of radioactive urea. The test is considered positive if carbon dioxide containing ^13^C is found in the second pair of samples [35].

UBT is a suitable test for diagnosing *H. pylori* infection in both adults and children aged between 3 and 11 years, with the condition that children should be administered a ‘test table’ containing 100 mL of orange juice [35,82,86,87,88]. Citrus juice is usually recommended to increase the contact time with the gastric mucosa by delaying gastric emptying. Moreover, this test might also be used for monitoring infection eradication at least 4 weeks after the completion of the eradication regimen [32]. Another major advantage of this test over serology or stool antigen is that it can be used in patients with a history of gastrectomy or those who recently received antibiotics or proton pump inhibitors [88] (Table 1). Nevertheless, the European Society of Pediatrics Gastroenterology Hepatology and Nutrition (ESPGHAN) recommends to wait at least 2 weeks after proton pump inhibitors and 4 weeks after antibiotics before performing this test in practice [85].

The specificity and sensitivity might even reach 100% [66,87]. In fact, a recent meta-analysis revealed that UBT might be used in children of any age but has lower sensitivity and specificity rates in those below the age of 6 years, 95% and 93.5%, in comparison with in children above this age, 96.6% and 97.7% [89] (Table 1).

False-positive results are uncommon, but they can occur in patients with a very recent history of endoscopy with a biopsy associated with gastrectomy or a significant alkalization of the gastric pH, but they can also be noticed when urea is hydrolyzed by other urease containing bacteria within the oral cavity or the stomach [90]. Therefore, the most recent ESPGHAN guideline stated that toothbrushing before the test in children might inactivate oropharyngeal bacteria, preventing false-positive results caused by this bacterial community [85]. The same effect was proven in the case of mouthwash with 1% chlorhexidine before taking this test [91], but its use in children remains controversial [85]. False-negative results are even less common than false-positive ones and are usually related to external factors such as method errors [54]. Other compounds and factors were incriminated as potential factors that could impair the result of breath test such as probiotics, which can alter the composition of the gut microbiota; prokinetics; diet, especially beans, potatoes, corn, wheat and oat flour; physical exercise, since the increased respiratory rate during exercise lead to a decrease in hydrogen; and cigarette smoking due to its associated increased excretion of hydrogen [85] (Table 1).

#### 2.1.6. Serology

A systemic immune response is triggered once *H. pylori* colonizes the gastric mucosa, resulting in the appearance of circulating anti-*H. pylori* antibodies after 3–4 weeks from infection. Despite the fact that all three types of antibodies—IgA, IgM and IgG—can be detected in the blood of an infected subject, only a validated IgG test is reliable based on the fact that *H. pylori* is a chronic infection [32]. Nevertheless, a study performed on pediatric subjects indicated that both IgG and IgA have a high accuracy in detecting this infection, especially in children below the age of 12 years [92]. Serology tests are non-invasive and widely available, have a low cost and do not require special equipment; they are therefore useful as screening tests. Three methods are available for detecting these antibodies: the enzyme-linked immunosorbent assay (ELISA), Western blotting and latex agglutination tests, but ELISA is the most frequently used [93]. Another major advantage of serologic tests is that they are not impaired by the recent administration of bismuth compounds, proton pump inhibitors, antibiotics, recent gastrointestinal hemorrhage or atrophic gastritis [32] (Table 1).

However, positive serology does not necessarily represent an acute infection since it is well-documented that antibodies to antigens can occur also as a result of a previous infection or due to non-specific cross-reacting antibodies [93]. Therefore, these tests are useful for primary diagnosis or for confirming another diagnostic test, but they should not be used for monitoring the eradication of *H. pylori* infection since it was additionally proven that quantitative antibody levels do not present a significant decline over a long period of time even after eradication. Another factor that might affect the reliability of serology is represented by the low prevalence of *H. pylori*, which can result in false-positive tests; therefore, the use a more reliable test such as cultures, histological tests, UBT or stool antigens are recommended in a population with a prevalence <40% [54,94] (Table 1). A current major concern worth discussing is represented by the increasing trends in immigration worldwide. Based on the differences in *H. pylori* antigenicity among different continents or even countries, due to the differences in prevalence, a standard test should be designed to eliminate this risk.

Based on all of the aforementioned facts, studies found a wide range regarding the specificity and sensitivity of serological tests, between 76% and 80% for sensitivity and between 79% and 90% for specificity [54,94].

#### 2.1.7. Stool Antigen Test (SAT)

SAT identifies the *H. pylori* antigen in stools, and it requires a small sample of feces that can be collected at home if it is sent to a laboratory within an appropriate time, where it can be preserved for a long time at −20 °C. If the sample is kept in improper conditions at room temperature for 72 h, the sensitivity of the test decreases to 69% [94]; otherwise, the test presents a sensitivity of 95.5% and a specificity of 97.6% [66,78]. Based on its good sensitivity and specificity rates, the SAT is appropriate for both primary diagnosis and eradication monitoring. In addition, SAT is a low-cost, easy-to-use and quick test, but it is not recommended in patients with acute diarrhea or watery stools [94] (Table 1).

Two types of SATs are currently available for *H. pylori* diagnosis, using either monoclonal or polyclonal antibodies: immunochromatography assay and enzyme immunoassay-based methods. However, the tests that used monoclonal antibodies present a higher accuracy when compared with polyclonal ones and are effective for detecting *H. pylori* infection in pediatric patients [94,95,96] (Table 1).

Similar to other tests mentioned above, SAT also requires a period of four weeks after antibiotics and bismuth and two weeks after the last use of proton pump inhibitors [94]. False-negative results were reported in several situations such as constipation, persistent gastrointestinal hemorrhage, low bacterial load or uniform distribution of antigen in the stool sample [94,96] (Table 1).

#### 2.1.8. Molecular Tests

As mentioned before, molecular tests can be invasive or non-invasive depending on the sample assessed. These methods are genetic-based methods that use PCR for detecting the DNA of *H. pylori* in gastric biopsy, feces, saliva or dental samples and have high specificity and sensitivity of up to 95%, but unfortunately, they are expensive and require highly-equipped laboratories and experienced staff [97]. The main advantage of these tests is that they are useful for detecting the virulence of *H. pylori* factors such as CagA and VacA involved in bacterial resistance (Table 1).

The assessment of gastric biopsy samples by molecular test is a laborious method that requires multiple specimens for culturing *H. pylori* and sequencing its DNA. Thus, multiple colonies of *H. pylori* must be picked for DNA extraction to obtain the most accurate identification of drug-resistant subpopulations. Eventually, the test will lead to the detection of multiple susceptible and resistant strains of *H. pylori* [68]. Gastric biopsy-based quantitative PCR was proven to have a higher accuracy than routine histology, culture or RUT alone in diagnosing pediatric *H. pylori* infection due to its ability of detecting low bacterial loads [98]. Contrariwise, the sequencing of DNA from stool samples is fast, providing results in less than 4 h; sensitive; and accurate and has lower costs. In addition, a smaller sample with fewer bacteria is required, and it does not require special equipment or special supplies for transportation. In addition, the usefulness of stool PCR was proven in pediatric patients with *H. pylori* for both targeting resistance-guided eradication therapies and for monitoring the emergence of clarithromycin resistance after the eradication regimen [99]. Nevertheless, the disadvantage of stool DNA PCR is related to the increased proportion of false-positive results, especially in recently treated patients due to the persistence in feces of coccoidal forms of *H. pylori*, which begin to decrease and fully disappear only at 8–12 weeks after successful eradication therapy [100] (Table 1).

A new approach in diagnosing *H. pylori* infection is represented by next-generation sequencing, which identifies mutations in genes associated with antibiotics resistance. Thus, based on these tests, multidrug-resistant strains might be detected in culture-negative biopsies by assessing mutations in the 23S rRNA, 16S rRNA and gyrA genes, with clarithromycin resistance being proven to be related to point mutations in nucleotide positions A2146 and A2147 of the 23S rRNA gene [78,101].

**Table 1:** Diagnostic tests for *H. pylori* infection.

Methods	Advantages	Disadvantages	Limitations
Endoscopy	*General endoscopy* visual assessment of gastric mucosa → identification of macroscopic abnormalities, suspect lesions and gastric biopsy samples [32] macroscopic nodularity → *H. pylori* infection and high mucosal density of the bacterium [36,37] linked color imaging and blue laser imaging [44,45] detection of both neoplastic and non-neoplastic lesions in the gastrointestinal tract [60].	invasive diagnostic method [32]	wide range of macroscopic aspects → different stages of gastritis varying from active inflammation and atrophy to intestinal metaplasia [35] interobserver or intraobserver variability augmented by the lack of objective indicators [56]
	*Narrow band imaging (NBI)* more specific endoscopy-based method → its capacity to provide a faster diagnosis [38] penetration variability of the light waves: → long wavelengths have the ability to penetrate deeper into the tissue, while short wavelengths remain superficial [38] useful for identifying the most-likely infected areas, indicating the optimal areas for biopsy [38] high correlation of this technique with the histopathological severity [53]	invasive diagnostic method [32] low specificity [38]	interobserver or intraobserver variability augmented by the lack of objective indicators [56]
	*Magnifying endoscopy* the prediction of *H. pylori* presence based on the assessment of the mucosal microvascular architecture [54] ‘pit plus vascular patters’ → significant sign for *H. pylori* infection [54] accurate when detecting *H. pylori* in both chromoendoscopy and white-light [55]	invasive diagnostic method [32]	interobserver/intraobserver variability augmented by lack of objective indicators [56] artificial intelligence-assisted endoscopy could be useful in providing a second opinion by avoiding operator dependency in diagnostic endoscopy [58,59]
Rapid urease test	low cost [66,67,68]. rapid and relatively highly specific detection of the presence of *H. pylori* after a maximum of 12–24 h [66,67,68] used for establishing the diagnosis of *H. pylori* infection and for monitoring eradication treatment [32] relatively specific test reaching up to 95–100% [66,68]	invasive test [66,67,68] false-negative results more common than false-positive ones and their exclusive use is not recommended for the exclusion of *H. pylori* infection [32]	moderately sensitive with a rate of 85–95% [66,68]
Histopatological exam	Giemsa stain → higher specificity and a lower false-positive rate [46] fluorescent nucleic acid peptide in situ hybridization → specificity of 100%, good cost-effectiveness, fast processing time → identification of the undetectable forms in routine staining [46,75,76,77] γ-glutamyl transpeptidase (GGT) achievable fluorescent probe → fast detection tool for *H. pylori* specificity of the histopathological exam can reach up to 100% [70,80] diagnosis of precancerous lesions [74]	Giemsa stain has a lower sensibility rate [46] fluorescent nucleic acid peptide in situ hybridization → the laborious preparation time, the need for a special microscope with fluorescence and an experienced observer [46,75,76,77] γ-glutamyl transpeptidase (GGT) achievable fluorescent probe → sensitivity remains poor, reaching only 82% [78] sensitivity varies between 50% and 95% depending on the location [70,80]	*H. pylori* can be detected at histopathological exam only on well-stained and sufficiently thin sections [74] some conditions → decrease the accuracy of histopathological exam (intestinal metaplasia, gastric atrophy, low bacterial density, uneven *H. pylori* distribution on the mucosal surface, long-term proton pump inhibitor use, antibiotics or bismuth administration) [54,74,79]
Culture	provides a wide spectrum of information regarding the morphological, biological and biochemical properties of *H. pylori* [66] determination of antibiotic resistance and its close monitoring [66] specificity of 100% [74,79] culture remains extremely valuable and according to the Maastricht V Consensus Report [32,82,83,84].	the sensitivity presents a wide variation, between 50 and 90% [74,79] false-negative results are possible due to several host-related and environmental factors [66]	laborious processing requiring strict transportation conditions for preserving the bacterium in a viable state, microaerophilic conditions with an oxygen content of less than 5%, high costs, designated nutrient media and experienced staff [54]
Urea Breath Test (UBT)	useful for diagnosing *H. pylori* infection in both adults and children aged between 3 and 11 years [35,82,86,87,88] monitoring infection eradication at least 4 weeks after the completion of the eradication regimen [32] can be used in patients with a history of gastrectomy or those who recently received antibiotics or proton pump inhibitors [88] the specificity and sensitivity might reach even 100% [66,87]	lower sensitivity and specificity rates in those below the age of 6 years, 95% and 93.5%, in comparison with that in children above this age, 96.6% and 97.7% [89] false-positive results → a very recent history of endoscopy with a biopsy associated with gastrectomy or a significant alkalization of the gastric pH, or if urea is hydrolyzed by other urease containing bacteria within the oral cavity or the stomach [90] false-negative results → related to external factors such as method errors [54]	potential factors that could impair the result of breath test such as probiotics; prokinetics; diet, especially beans, potatoes, corn, wheat and oat flour; physical exercise; and cigarette smoking [85]
Serology	non-invasive, widely available, low-cost and does not require special equipment for a screening test three methods: the enzyme-linked immunosorbent assay (ELISA), Western blotting and latex agglutination tests, with ELISA being the most frequently used [93] not affected by the recent administration of bismuth compounds, proton pump inhibitors, antibiotics, recent gastrointestinal hemorrhage or atrophic gastritis [32] useful for primary diagnosis or for confirming another diagnostic test [54,94]	positive serology does not represent an acute infection [93] should not be used for monitoring the eradication of *H. pylori* infection [54,94] false-positive results → low prevalence of *H. pylori* [54,94]	sensitivity between 76% and 80%, and specificity between 79% and 90% [54,94]
Stool antigen test (SAT)	monoclonal antibodies present a higher accuracy when compared with polyclonal ones and are effective for detecting *H. pylori* infection in pediatric patients [94,95,96]	requires a period of four weeks after antibiotics and bismuth and two weeks after the last use of proton pump inhibitors [94]	several conditions affect the test: constipation, persistent gastrointestinal hemorrhage, low bacterial load or uniform distribution of antigen in the stool sample [94,96]
Molecular tests	*Invasive molecular tests* detect DNA of *H. pylori* in gastric biopsy → high specificity and sensitivity of up to 95% [97] useful for detecting the virulence *H. pylori* factors such as CagA and VacA involved in bacterial resistance [97] gastric biopsy-based quantitative PCR → higher accuracy that routine histology, culture or RUT alone in diagnosing pediatric *H. pylori* infection → ability of detecting low bacterial loads [98]. the next-generation sequencing identifies mutation in genes associated to antibiotics resistance → these tests multidrug resistant strains might be detected in culture negative biopsies by assessing mutation in the 23S rRNA, 16S rRNA and gyrA genes [78,101]. *Non-invasive molecular tests* detect DNA of *H. pylori* in feces, saliva or dental samples → high specificity and sensitivity of up to 95% [97] useful for detecting the virulence *H. pylori* factors such as CagA and VacA involved in bacterial resistance [97] sequencing of DNA from stool samples is fast, providing the results in less than 4 h; sensitive; and accurate and has lower costs [99] stool PCR → in pediatric patients with *H. pylori* for both targeting resistance-guided eradication therapies and monitoring, the emergence of clarithromycin resistance after the eradication regimen [99]	expensive and requires highly equipped laboratories and experienced staff [97] laborious method that requires multiple specimens for culturing *H. pylori* and sequencing its DNA [68] expensive and requires highly equipped laboratories and experienced staff [97] false-positive results → in recently treated patients due to the persistence in feces of coccoidal forms of *H. pylori*, which begin to decrease and fully disappear only at 8–12 weeks after successful eradication therapy [100].	the high costs of the procedures

## 3. Conclusions

The early diagnosis of *H. pylori* infection is crucial for preventing the associated complications, especially, the onset of carcinogenesis processes. Thus, the detection of this bacterium during childhood might represent the cornerstone in terms of *H. pylori*-associated gastric cancer prophylaxis. Without a proper diagnosis, no proper eradication is possible, and therefore, the choice of diagnostic tool should be targeted based on the patient’s characteristics. Non-invasive diagnostic tools such as endoscopy, histology, culture or biopsy-based PCR are definitely more accurate than non-invasive ones, but they should remain the last option only for a small group of patients since the current medical era focuses more and more on non-invasive diagnostic approaches associated with lower discomfort for patients. Nevertheless, non-invasive diagnostic methods should be selected based on both their accuracy and their associated costs. Among the non-invasive tests, probably the best choice for diagnosing pediatric *H. pylori* infection is the urea breath test and stool antigen. Moreover, molecular non-invasive techniques can strengthen the diagnosis in select cases.
